# Poverty Dynamics in Early Childhood among the Native-Born Children of Immigrants in Sweden and Finland

**DOI:** 10.1007/s10680-026-09772-y

**Published:** 2026-03-25

**Authors:** Raffaele Grotti, Siddartha Aradhya, Maria Vaalavuo, Outi Sirniö

**Affiliations:** 1 University of Trento, Trento, Italy; 2 Stockholm University Demography Unit (SUDA), Department of Sociology, Stockholm University, Stockholm, Sweden; 3 Finnish Institute for Health and Welfare (THL), Helsinki, Finland; 4 Institute for Analytical Sociology (IAS), Department of Management and Engineering, Linkööping University, Norrkööping, Sweden

**Keywords:** Childhood poverty, Sweden, Finland, Poverty dynamics, Poverty persistence, Immigration

## Abstract

This brief report delves into the poverty dynamics of second-generation immigrant (G2) children in Sweden and Finland, comparing them with their peers from the majority population. These are unique contexts to examine such socioeconomic inequalities since they are the two highest ranked countries in terms of the Migrant Integration Policy Index. This study draws on longitudinal full-population register data from 2011–2019 for children aged 0–4. This study contributes to existing research by providing a detailed, up-to-date analysis of socioeconomic disparities between a wide range of second-generation immigrant groups and majority children during early childhood—a period widely recognized as critical for development and strongly influenced by work-family reconciliation and family welfare policies. Findings reveal significant inequalities with G2 children facing longer exposure to poverty, higher poverty persistence and higher poverty entry rates than majority children. Interestingly, the G2’s longer exposure to poverty is not due to a higher likelihood of remaining poor, but rather a greater risk of falling into poverty. The study underscores the severe poverty levels among some immigrant groups, G2 Somalis being the chief example, as a critical challenge to equality and social stability in both countries.

**Supplementary Information:**

The online version contains supplementary material available at 10.1007/s10680-026-09772-y.

## Introduction

As European countries grapple with the complexities of increasingly multicultural societies, understanding the socioeconomic outcomes of immigrants and their descendants is critical. Sweden and Finland, often lauded for their progressive welfare policies that promote economic equality, provide a unique context to examine the extent to which countries with strong social policies are faring in terms of social inequality and immigrant integration. To this end, this article examines early childhood income poverty among the children of Swedish and Finnish born parents (hereafter the majority population) and the children born in Sweden and Finland to immigrant parents (hereafter the G2).

The focus on socioeconomic inequalities between the G2 and majority population in Sweden and Finland provides an opportunity to explore nuances between two similar yet distinct contexts. The countries are ranked as the two most favorable countries in the world in terms of policies promoting the integration of immigrants according to the MIPEX index (Solano & Huddleston, 2020). Specifically, they employ comprehensive approaches to integration that fully guarantees equal rights, opportunities, and security for immigrants. This is in large part due to the existence of generous welfare states focused on combatting socioeconomic inequalities through a variety of family policies and services.

At the same time, the countries differ in their experiences with migration. Sweden has experienced large-scale migration since the late 1960s, whereas Finland has experienced net immigration only from the 1990s. This is also visible in the share of the foreign-born populations and their descendants, as well as the diversity of origin groups, which is far more pronounced in Sweden. Moreover, the two countries have diverged in more recent years in their poverty and inequality trends as both have increased in Sweden while they have remained more or less stable in Finland that ranks as one of the best performers in terms of child poverty in Europe (UNICEF Innocenti 2023).

Childhood poverty is a key societal-level indicator of immigrant integration since it reflects the consequences of inequalities in the labor market and the effectiveness of social transfers to mitigate socioeconomic disadvantages. Experiences of childhood poverty are linked to a wide range of socioeconomic and health outcomes throughout the life-course and over generations (Duncan et al., 1998; Evans, 2016; Parolin et al., 2022). Recent evidence suggests that the consequences of poverty may be more pronounced for migrants in terms of developmental delay and school readiness when experienced at younger ages (Gill et al., 2024). As such, the degree to which experiences of childhood poverty are unequally distributed between the children of immigrants and the majority population can inform projections of future developments of inequality in the host society.

The current study builds on existing research on poverty among immigrants by applying a longitudinal perspective for a number of relevant G2 groups. Our main goal is describing whether and to what extent G2 children aged 0 to 4 years old are disadvantaged compared to majority children in terms of poverty.

This brief report answers the following research questions: (1) What are the poverty experiences of G2 children as compared to majority children in Sweden and Finland? Do (2) the length of poverty exposure and (3) year-to-year poverty transitions differ across groups?

Question (2) refers to the total number of years that children spend in poverty over the first five years of life. Question (3) examines how experiencing poverty in one year increases the probability of experiencing it again in the following year. Distinguishing between these two is important in order to understand “longitudinal” poverty from a descriptive perspective, as well as the processes contributing to them. More specifically, whether poverty exposure is the result of persistence.

We address these research questions by examining poverty rates and poverty transitions in the first five years of life for cohorts born between 2011 and 2015 (followed between 2011 and 2019). To do this, we exploit full-population register data.

We contribute to existing research by 1) exploring the experience of childhood poverty in two Nordic welfare states with good records of both immigrant integration and family investment policies; 2) focusing on a vulnerable early life stage during which family policies aim at reducing poverty and facilitating work-family reconciliation; and 3) providing a dynamic description of poverty among specific immigrant groups. The availability of register data makes the study unique, because survey data grants only limited opportunities to analyze poverty dynamics among immigrant families due to small sample sizes. More specifically, we examine migrant groups at a high level of granularity and provide a longitudinal and up-to-date (including more recent arrivals and the largest immigrant groups in these countries today) picture of children born in Sweden and Finland.

## Literature

### Poverty Inequality Across Ethnic/Minority Groups

Existing literature has shown that poverty is an experience of varying duration. While for the majority of people poverty spells are transient or of short duration, most poverty-years are experienced by a small group for whom poverty is chronic (Bane & Ellwood, 1986; Layte & Whelan, 2003; Mood, 2015). This pattern has also been observed for childhood poverty (Lindquist & Sjögren Lindquist, 2012; Ratcliffe & McKernan, 2010). Importantly, the negative consequences of poverty are more severe if poverty persists (UNICEF Innocenti 2023).

Additionally, poverty is not equally distributed across socio-demographic groups, such as immigrants (Fouarge & Layte, 2005; Parolin et al., 2022). Existing research in Sweden and Finland has shown that children of immigrants are at a higher risk of experiencing income poverty. Concerning Sweden, Gustafsson and Österberg (2018) found large variation in poverty rates between native and immigrant children between the ages of 0 and 17.

Moreover, immigrant and ethnic minority groups are not only more likely to experience poverty, but they are more at risk of remaining in poverty. For example, the prevalence and persistence of poverty in the US was strongly concentrated among African-American children and immigrants with Hispanic backgrounds (Corcoran & Chaudry, 1997; Ratcliffe & McKernan, 2010; Thiede et al., 2021). This pattern has also been observed in Sweden and Finland (Galloway et al., 2015; Gustafsson & Österberg, 2018; Lindquist & Sjögren Lindquist, 2012; Obućina & Ilmakunnas, 2020).

## The Experience of Poverty Over the Life Course

Individuals may experience long exposure to poverty because they have characteristics that make them susceptible. Common risk factors include family structure, education, and labor market status, where single-mothers and large families, low-educated, and unemployed individuals are particularly vulnerable (Biewen, 2014; Zagel et al., 2022). To the extent that these characteristics persist over time, they increase poverty risks in both current and future periods. Immigrants in Sweden and Finland are generally more likely to fall into most of these categories compared to the majority population, making them particularly susceptible. Importantly, poverty due to these characteristics might be relatively limited in Sweden and Finland than other countries because of generous welfare policies which support disadvantaged individuals.

In addition, individuals may experience long poverty exposure due to poverty persistence. Poverty persistence may be the result of people having characteristics which make them poverty prone as discussed above; however, poverty can also persist over time independently by triggering a set of behavioral changes that increase the risk of experiencing poverty repeatedly (Biewen, 2009, 2014). This process is often referred to as genuine state dependence thus representing an independent mechanism driving long-term poverty. Given that social exclusion is a salient feature of the migrant experience, which is embodied by high levels of discrimination in the labor market, one can assume that experiencing poverty may exacerbate processes related to genuine state dependence among immigrants.

Beyond individual characteristics and behavioral mechanisms, the timing of poverty experiences across the life course also matters for understanding immigrant-native inequality. Early childhood and becoming a parent have been identified as phases of heightened poverty risk according to traditional theories on poverty cycles over the life course (Rowntree, 1902). On the one hand, the challenges of reconciling work and family life during this period affect both majority and immigrant populations, potentially creating a life stage where poverty risks converge more than at other points in the life course. On the other hand, immigrants can be double burdened around childbirth if they do not meet the eligibility criteria for social transfers or receive only minimum benefits, putting them at a higher poverty risk in this life phase.

## Study Contexts

Sweden and Finland have undergone important demographic shifts as a result of immigration over recent decades, though their trajectories differ significantly. In the 1990s, Finland shifted from a country of emigration to one of immigration. While the immigrant population remains modest in size compared to its Nordic neighbors, it has grown tenfold over the last 30 years—today around eleven percent of the overall population is foreign-born or born in Finland with parents born abroad.

Sweden experienced a similar development earlier, beginning in the 1970s when immigration from outside Europe came to dominate immigration flows. The share of Sweden's population born outside the country (G1) or born in Sweden with at least one parent born abroad (G2) continues to increase, today representing 30 percent of the entire resident population (Aradhya and Mussino 2020).

Research on immigrant integration in both countries largely confirms patterns typical of other European countries. Immigrants have lower labor force participation, and even when economically active, they face higher unemployment rates and lower earnings than comparable majority natives (e.g., Aradhya, Grotti, and Härkönen 2023; Carlsson and Rooth 2016; Sarvimäki 2011). These native-foreign gaps tend to persist long after immigration, with considerable heterogeneity between immigrants by country of origin (Vaalavuo and Rask 2024). Importantly, certain immigrant groups face more restrictive structural and cultural barriers that impede their integration (Nshom, Sadaf, and Ilkhom 2022; Quillian et al., 2019).

Despite these integration challenges, a key feature of both contexts is their strong welfare structures aimed at supporting vulnerable populations. While social policy has significantly altered the traditional profile of poverty and created important cross-national variation in childhood poverty (Kangas and Palme 2000), both Sweden and Finland share a commitment to supporting families. Both countries offer several benefits targeted to parents of small children to compensate for income loss due to childbirth, facilitate parental labor market participation, and provide income support through non-contributory child benefit payments.

However, these welfare provisions may paradoxically generate inequality. Immigrants are more likely to receive minimum rather than earnings-related benefits with work history requirements, and face additional obstacles in accessing the labor market despite heavily subsidized childcare facilities. Research on parental leave policies has shown how policy design affects immigrant and native-born families differently (Tervola, Duvander, and Mussino 2017). Comparing Finland and Sweden, Tervola et al. (2017) found that Sweden’s more flexible parental leave system with stronger earmarking (father’s quota) led to a higher take-up among immigrant fathers and narrower immigrant-native gaps. In contrast, Finland’s introduction of parental leave reforms primarily increased take-up among native-born fathers and immigrants from Western countries, while other immigrant groups showed no increase—likely due to information deficits and smaller immigrant networks. Tervola (2018) has similarly shown that both cultural norms and policy context affect childcare choices in Finland and Sweden, with immigrants' choices distinct from those of the majority.

Current political trends further complicate this picture. Both countries show tendencies to exclude immigrants from certain family benefits—for example, the care allowance for small children in Finland and discussions about benefits ceilings for large families in Sweden.

## Data and Methods

Analyses are based on Finnish and Swedish total population registers covering all the individuals residing in the countries.^1^ Our target population consists of children born between 2011 and 2015 who are followed for 5 years from birth until age 4. We included only children who we were at least able to observe consecutively from age 0 to 2. As we are using full-population register data the only reasons for attrition are death and emigration. As a result, we observe 563,404 children born in Sweden (for a total of 2,809,661 person-year observations) and 285,423 children born in Finland (1,429,665 observations). Table 1 illustrates the data structure for the two countries.

**Table 1:** Data structure and number of observations for Sweden and Finland

Sweden				Age			
		0	1	2	3	4	Total
	2011	110,663					110,663
	2012	111,947	110,663				222,610
	2013	112,361	111,947	110,663			334,971
Year	2014	114,188	112,361	111,947	110,072		448,568
	2015	114,245	114,188	112,361	111,367	109,552	561,713
	2016		114,245	114,188	111,876	110,930	451,239
	2017			114,245	113,738	111,428	339,411
	2018				113,818	113,306	227,124
	2019					113,362	113,362
	Total	563,404	563,404	563,404	560,871	558,578	2,809,661

Finland				Age			
		0	1	2	3	4	Total
	2011	58,919					58,919
	2012	58,496	59,120				117,616
	2013	57,222	58,682	59,262			175,166
Year	2014	56,514	57,428	58,809	59,112		231,863
	2015	54,272	56,678	57,534	58,626	58,921	286,031
	2016		54,436	56,806	57,366	58,492	227,100
	2017			54,572	56,604	57,230	168,406
	2018				54,267	56,295	110,562
	2019					54,002	54,002
	Total	285,423	286,344	286,983	285,975	284,940	1,429,665

Our outcome is relative income poverty. We focus on relative rather than absolute poverty as it captures social exclusion to a greater extent, an aspect that is particularly relevant when considering migrants. Specifically, relative poverty is closely related to economic inequality within a society, identifying individuals who fall significantly below the average living standards of that society, even if their basic needs are met. We use the standard poverty measure of the European Union in which children are categorized as income poor if their family’s equivalized disposable household income falls below the threshold set at 60% of population median income. We defined children’s immigrant background according to their mother’s country of birth. We distinguish origins corresponding to the largest as well as the most distinct groups – see Table 2 and 3 for Sweden and Finland respectively.

**Table 2:** “Snapshot” poverty and poverty length of exposure, Sweden. Children between ages 0 and 4

	Group size	Snapshot poverty	Poverty length of exposure (N. of years in poverty between age 0–4)						
	%	Age 0–4	0	1	2	3	4	5	Total
Sweden	74.16	10.51	79.71	7.05	4.14	2.93	2.48	3.68	100
All G2	25.84	42.91	39.46	9.36	8.45	8.72	9.30	24.72	100
Finland	0.5	12.93	76.43	7.74	4.17	3.71	3.07	4.88	100
Other EU	2.14	22.41	65.18	7.64	5.85	5.72	4.75	10.86	100
Other	1.29	23.08	60.14	10.51	7.38	7.10	6.04	8.83	100
Other Asia	3.48	30.35	52.20	10.28	8.24	7.41	7.65	14.21	100
East EU	4.72	30.38	51.01	11.03	9.03	7.71	7.20	14.03	100
USSR-Russia	1.33	32.55	49.21	10.01	8.78	9.08	7.63	15.29	100
Other Middle-East	2.5	47.63	33.77	9.34	9.00	9.89	10.33	27.68	100
Turkey	0.81	48.09	30.02	11.58	10.27	11.17	11.56	25.40	100
Other Africa	2.59	51.54	26.99	10.72	10.50	10.96	12.43	28.41	100
Iraq	3.16	61.27	19.86	8.53	9.24	10.82	13.04	38.51	100
Syria	1.06	64.17	17.94	7.26	8.63	11.03	13.10	42.04	100
Somalia	2.26	79.17	7.18	5.17	6.54	8.75	12.20	60.15	100
Total	100	18.88	69.27	7.65	5.26	4.43	4.25	9.14	100

“Snapshot” poverty rate mimics a cross sectional rate where poverty is computed for all ages 0–4 pooled. Data sorted by “snapshot” poverty rate. N. children 563,404. N. observations 2,809,661

**Table 3:** “Snapshot” poverty and poverty length of exposure, Finland. Children between ages 0 and 4

	Group size	Snapshot poverty	Poverty length of exposure (N. of years in poverty between age 0–4)						
	%	Age 0–4	0	1	2	3	4	5	Total
Finland	90.21	11.56	74.88	9.91	5.85	4.02	2.86	2.47	100
All G2	9.79	35.17	37.37	16.08	13.57	12.02	10.17	10.79	100
Estonia	1.11	23.73	51.45	16.71	11.96	8.78	6.39	4.71	100
Sweden	0.34	21.53	56.32	14.45	11.13	7.72	6.02	4.36	100
Other Asia	1.65	22.57	57.34	13.33	8.70	8.12	5.86	6.65	100
USSR/Russia	1.81	25.26	52.75	13.63	10.67	8.43	7.33	7.19	100
Other	2.60	26.55	50.53	14.31	11.16	9.07	7.32	7.61	100
Other Africa	0.85	34.93	36.51	16.59	14.55	13.02	9.22	10.12	100
Turkey	0.24	49.37	21.06	14.43	15.15	14.31	17.46	17.60	100
Iraq	0.41	51.23	16.08	15.87	17.47	17.69	15.43	17.47	100
Somalia	0.77	51.49	15.61	16.92	16.14	17.13	15.69	18.51	100
Total	100	13.31	72.07	10.39	6.44	4.63	3.40	3.08	100

“Snapshot” poverty rate mimics a cross sectional rate where poverty is computed for all ages 0–4 pooled. Data sorted by “snapshot” poverty rate

## Analytical Strategy

We provide results related to four descriptive measures of poverty. First, we show *“snapshot” poverty* rates. That is roughly equivalent to a cross-sectional rate where poverty is computed for the entire set of person-year observations, thus pooling all children for the entire observational window. Second, we present *poverty length of exposure* measured as the total number of years poverty is experienced from age 0 to 4. This measure encompasses concepts including transient, recurrent and chronic poverty that are sometimes used in the literature (Bane & Ellwood, 1986; Fouarge & Layte, 2005; Lindquist & Sjögren Lindquist, 2012; Obućina & Ilmakunnas, 2020). Third, we provide the persistent poverty transition rate that is measured as the probability of being poor (time *t*) for those who were poor in the previous year (time *t-1*). This is a commonly used metric of state dependence in poverty research, and here we reference to that literature (Ayllón & Gábos, 2017; Biewen, 2009). Length of exposure and persistence rates represent longitudinal measures of poverty but emphasize distinct aspects of poverty dynamics. Persistence focuses on short-term, year-to-year, dynamic of poverty; length of exposure focuses on the longer-term dimension, in terms of accumulation over time of the poverty experience. Importantly, the experience for some groups of higher persistence implies also higher accumulation of poverty in the long run – e.g., 5 years of exposure could be the result of families entering poverty and not being able to escape. Finally, we present the poverty entry transition rate that is measured as the probability of being poor (time *t*) for those who were not poor in the previous year (time *t-1*). This dynamic is likely to contribute in accounting for poverty length of exposure, where the higher the risk of entering into poverty and the longer the exposure to poverty.

We present unadjusted results as opposed to results adjusted for compositional factors in order to more accurately illustrate the real-world scenario. We argue that this is a useful starting point to understand the effectiveness of Sweden’s and Finland’s immigrant integration policies. An analysis adjusting for compositional differences would dilute this perspective since the variables one would control for are the result themselves of immigrant origin. For example, adjusting for family size—a factor that is associated to poverty—would lead to a comparison of majority and minority groups with the same family size. In a real-world scenario, however, some groups have larger families on average, for example the Somali population. This renders a comparison after adjustment unrepresentative of inequalities in poverty experiences across groups, contrasting with the aims of the study. However, to understand any notable differences in the background characteristics between natives and immigrants in Sweden and Finland, we provide this information in the Appendix Table A1 and A2.

## Results

### Snapshot Poverty

Table 2 and Table 3 describe the group differences in childhood poverty for Sweden and Finland, respectively. In Sweden, roughly one in four children (26%) have immigrant parents. The overall snapshot poverty rate is 18.9 percent. When comparing the G2 to majority children we see large differences with the former displaying a 32-percentage point higher poverty rate, at 42.9 percent. This figure, however, masks significant disparities across groups. For instance, the poverty rate of children of Somali descent is markedly high at 79.2 percent (snapshot poverty). This contrasts sharply with the snapshot rate for majority children, which is the group with the lowest levels of poverty at 10.5 percent. There are notable differences also between Middle-Eastern countries, ranging from 48.1 for Turkey to 64.2 percent for Syria. G2 from European countries also experience higher levels of snapshot poverty as compared to majority Swedes, with G2 Finns beingthe group with the closest poverty rate to the majority population, at 12.9 percent.

In Finland (Table 3), the G2 represent a much smaller share (about 10%) as compared to Sweden. The overall snapshot poverty rate was 13.3 percent, noticeably lower than in Sweden.^2^ The rate for majority children is 11.6 percent while for G2 children it is 29.5 percent. Snapshot poverty for majority children in Finland and Sweden is comparable, highlighting similar economic standing for majority populations in both countries. However, G2 Swedes in Finland have a significantly higher poverty rate (21.5%) than majority Finns (11.6%) or than children G2 Finns in Sweden (12.9%). The G2 display much lower poverty rates in Finland than in Sweden.

Focusing on similar G2 groups in both countries highlight distinct patterns. The groups with the highest snapshot poverty rates in Finland are G2 Turks, Iraqi, and Somali children who have similar snapshot poverty rates ranging between 49 and 51 percent. These groups are also among those that display the highest snapshot poverty rates in Sweden However, differences between these groups are remarkable. For example, G2 Turks display snapshot poverty rates of 48 percent, G2 Iraqi 61 percent, and G2 Somali 79 percent in Sweden. Interestingly, G2 Turks have similar poverty rates in both Finland and Sweden; however, G2 Iraqi and Somali children are far more disadvantaged in Sweden.

## Poverty Length of Exposure

In Table 2, majority Swedes experience the lowest length of exposure across all groups with only 3.7 percent experiencing five years of poverty in the first five years of life, and 79.7 percent experiencing zero years of poverty. The G2, in general, experience significantly longer poverty. Only roughly 40 percent of the G2 experience no poverty in their first five years of life whereas nearly one out of four is poor for the entire period. When turning to specific origin groups, we see large variation. Notably, 60.2 percent of G2 Somali children experience five consecutive years of poverty and only 7.2 percent experience zero years of poverty.^3^ To put it differently, Somali children are 16 times more likely to experience five years of poverty as compared to majority Swedes (in the case of snapshot poverty, the former is “only” eight times more likely to experience poverty as compared to the latter).

In Table 3, among majority Finns, 74.9 percent experience zero years of poverty while 2.5 percent experience five years of poverty in the first five years of life. Less than half of the G2 experience no years of poverty and 8.7 percent experience five years of poverty. Again, as in Sweden, there is large variation across origin groups albeit to a smaller extent. For example, among G2 Somali children, only 15.6 percent experience zero years of poverty, while almost a fifth (18.5 percent) live in poverty for the entire early childhood.

Directly comparing the number of years spent in poverty in the two countries reveals nuanced differences. The overall lower poverty rates in Finland suggest a more favorable economic environment for G2 children compared to Sweden. Specifically, in Sweden, 69.3 percent of children do not experience poverty, while 9.1 percent experience poverty for all five years. In contrast, in Finland, these numbers are slightly more favorable, with 72.0 percent of children experiencing no poverty and only 3 percent facing it for all five years. The comparison of group-specific poverty exposure between the two countries reveals differences that are even more pronounced. For example, the five-year poverty exposure for G2 Somalis is staggeringly high in Sweden (60.2 percent) as compared to Finland (18.5 percent). The same pattern is observed among G2 Iraqi children with five-year poverty exposure at 39 percent compared to 17.5 percent in Finland. Among the G2 Turks, although snapshot poverty rates are slightly lower in Sweden, they experience higher five-year poverty (roughly 25 percent) as compared to Finland (18 percent).. In general, the G2 in Finland have shorter exposure than their counterparts in Sweden, with five-year poverty occurring three times as often among G2 in Sweden.

## Poverty transitions

**Fig. 1:**
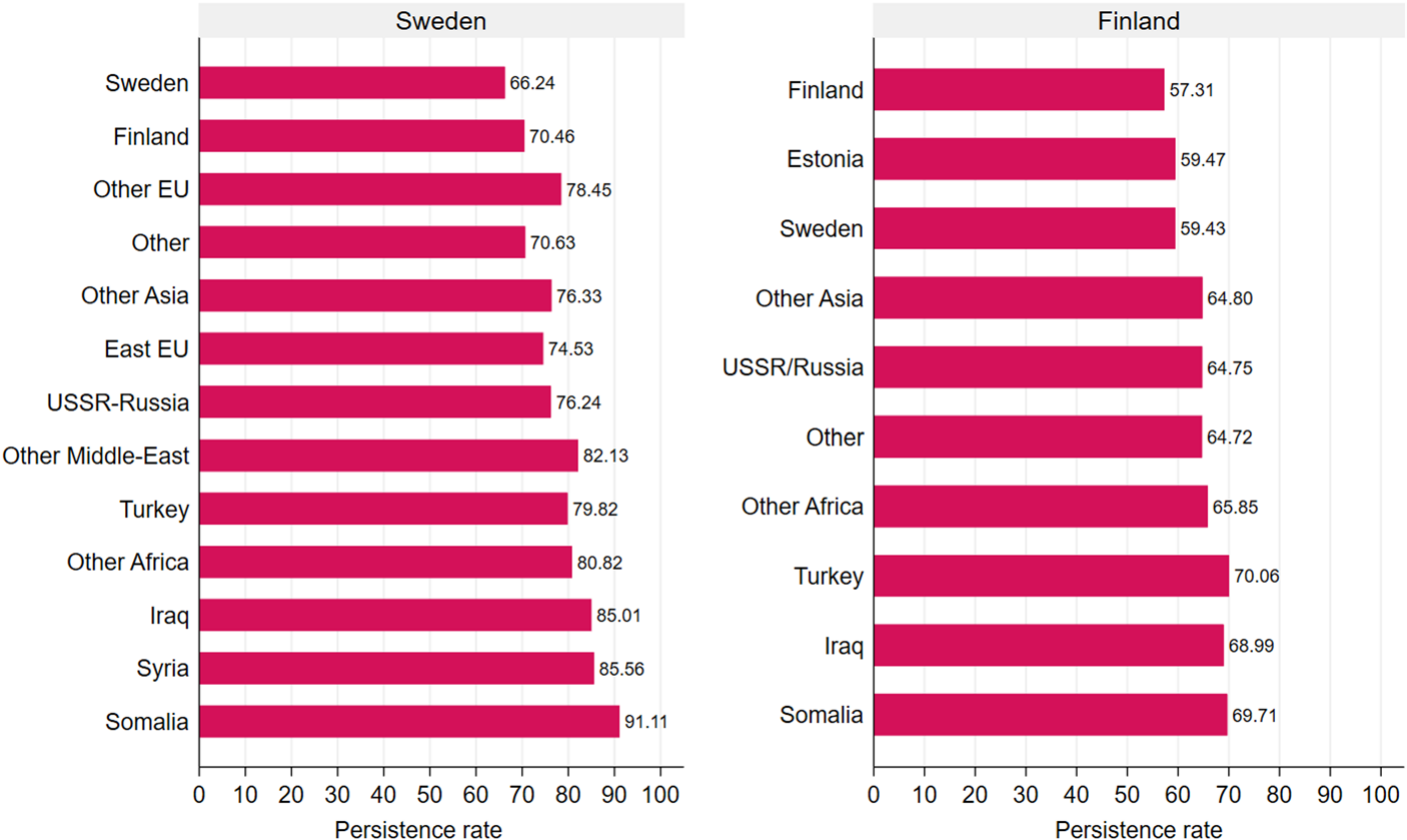
Persistence childhood poverty rate, Sweden and Finland. Probabilities sorted by “snapshot” childhood poverty rate

The next analyses allow us to understand whether longer exposure is reflected in persistence, where the former is the result of the latter. Figure 1 presents descriptive poverty persistence rates for both Sweden and Finland. Majority Swedes display the lowest poverty persistence rate at 66.2 percent. This means that roughly two out of three majority Swedes in poverty remain poor. This is in contrast to G2 Somali children who show a persistence probability which is slightly above 91 percent. Although we observe large inequalities in the persistence rates, they are much less striking than the group differences observed in Table 2. Turning to the results for Finland, we see that majority Finns display a poverty persistence rate of 57.3 percent. G2 Estonians (59.5%) and G2 Swedes (59.4%) are relatively close to this rate. The groups with the highest poverty persistence rates are the G2 Turks (70%), G2 Somalis (69.7%) and G2 Iraqis (69.0%). Although there remains inequality in poverty persistence, it is of a slightly smaller magnitude as compared to that observed in Sweden.

When comparing Sweden and Finland in persistence rates, Sweden displays higher persistence rates overall ranging from 66 to 91 percent as compared to Finland from 57 to 70 percent. When comparing specific groups, we also see stark differences. G2 Turks and Somalis in Finland experience 10 and 20 percentage point lower persistence rates, respectively, as compared to their counterparts in Sweden.

**Fig. 2:**
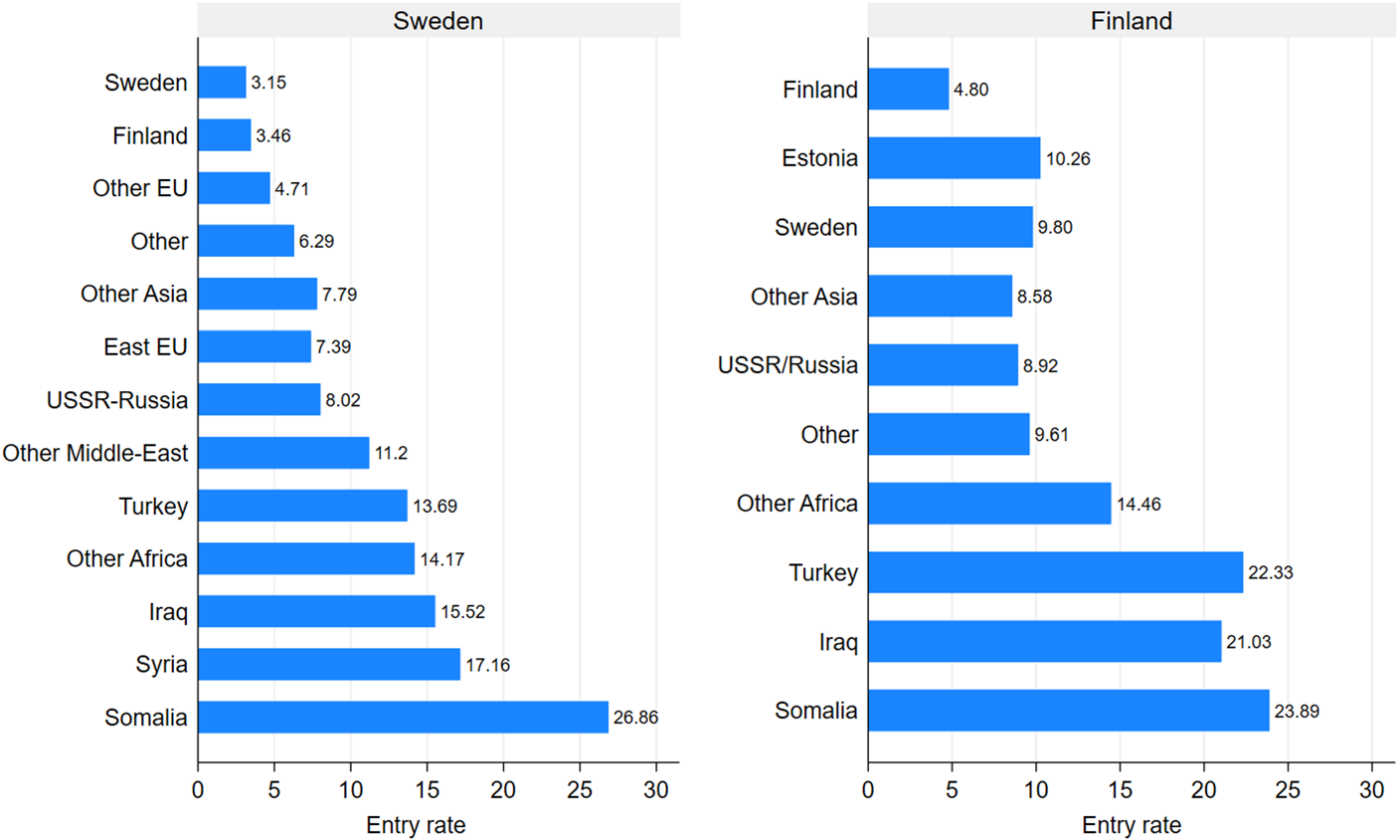
Entry childhood poverty rate, Sweden and Finland. Probabilities sorted by “snapshot” childhood poverty rate

Our results so far have importantly shown that inequalities in poverty persistence rates are significantly lower than the inequalities observed in poverty length of exposure in both countries. This raises the question: If poverty persistence does not explain group differences in poverty exposure, what does? In order to address this question, we complement our findings with poverty entry rates shown in Fig. 2. In Sweden, among the children of Swedish-born parents, 3.2 percent of individuals who were not in poverty in the previous year became poor (entered) in the following one. This figure is significantly lower than many other groups, apart from G2 Finns. For example, the poverty entry rates range from 4.7 percent for G2 with Other EU origins to as high as 26.9 percent among G2 Somalis. With respect to the latter, this tells that more than one in four children with Somali-born parents became poor when they were not in poverty in the previous year. In Finland, we see a similar pattern with the children of Finnish-born parents experiencing an entry rate of 4.8 percent. All other groups experience higher entry rates ranging from 8.6 percent among G2 from Other Asia to 23.9 percent among G2 Somalis. As one can see, we observe much more variation across groups when considering poverty entry rates than persistence rates.

Interestingly, and in contrast to the previous findings where Sweden displayed higher overall poverty figures (“snapshot”, length of exposure, and persistent rates), the majority children in Finland experience a 1.7 percentage point higher entry rate as compared to the majority children in Sweden. The average poverty entry rate in Sweden was 4.3 percent whereas in Finland entry rates were 5.3 percent. In other words, children born in Finland are more likely to enter poverty as compared to children born in Sweden. To further examine potential reasons for this, we analyzed distance to poverty threshold both among poor families and non-poor families with income below the median (Figure 1 in the Appendix). These additional results show that families in Finland have incomes closer to the poverty threshold than families in Sweden, making it more likely for the former to fluctuate in and out of poverty with smaller changes in their income.

## Discussion

Socioeconomic outcomes for the descendants of immigrants can be considered a key indicator for inclusiveness of our societies and social sustainability. In this study, we described longitudinal poverty experiences of children aged 0 to 4 years old with native and foreign background in two Nordic welfare states. Notwithstanding Sweden and Finland lead the international ranking as the two most favorable countries in the world in terms of policies promoting the integration of immigrants, we register large inequalities across origin groups in childhood poverty, especially with respect to the most severe situation: longer exposure to poverty. Interestingly, our results show that child poverty rates among majority children do not significantly differ between the two countries, but the diverging trends in recent years could be driven by larger shares of immigrant children in Sweden. Consequently, to understand developments in poverty and to design anti-poverty measures, we must consider poverty in conjunction with immigration.

The results are in line with what we know about poverty dynamics. Poverty is a transitory experience for the majority of people (e.g., majority children), while for a minority, poverty is a long-term experience (e.g., Somali children). Such long exposure to poverty is likely to have negative effects on children’s later outcomes, hampering the ideal of equal opportunities celebrated in both Sweden and Finland. Similar inequalities are visible in both countries, but the magnitude of differences is larger in Sweden. Our analysis on poverty persistence and entry complemented the picture.

The finding that inequalities are larger in Sweden than in Finland may reflect differences in migration histories. Segmented assimilation theory suggests that immigrants experience divergent integration trajectories depending on context of reception, human capital, and community resources (Portes and Zhou 1993). Sweden’s longer migration history has possibly allowed these divergent pathways to become institutionalized.

Sweden’s larger immigrant population has produced high degrees of residential segregation (Aradhya et al., 2017; Haandrikman et al., 2023). While providing social support for some, these residential patterns can limit broader integration opportunities and labor market access for others (Musterd & Andersson, 2006). Additionally, immigrants face substantial labor market discrimination, particularly those from Middle Eastern and African origins (Quillian et al., 2019). It is possible that multiple immigration waves have created ethnic niches that channel certain groups into disadvantaged positions, perpetuating inequality across cohorts.

Finland’s recent transition to an immigration country may suggest that such patterns are still emerging. Without policy interventions, similar segmented assimilation processes may develop as socioeconomic stratification crystallizes and the immigrant population grows. It is worth mentioning that there may be different selection processes in who stays and becomes a parent in Sweden and in Finland. With longer immigration history and stronger networks, it is possible that those with weaker economic opportunities remain in Sweden, while similar migrants choose to emigrate from Finland.

It is important to note that the immigrant groups that display high levels of poverty and persistence, likely have similar reasons for migration. Somali and Iraqi populations in Finland and Sweden generally arrived as asylum seekers, whereas immigrants from Turkey were more mixed. These differences in reason of migration likely shape the socioeconomic disadvantages these families face in the host countries, leading the former two groups to experience the highest levels of disadvantage; but it does not explain the differences between Finland and Sweden.

Other factors contributing to greater inequality in Sweden compared to Finland may be linked to labor market institutions. For instance, Sweden enforces last-in, first-out rules by law, whereas such regulations do not exist in Finland. Under this rule, workers are dismissed based on their seniority. Since immigrants often have shorter tenures, they are at a higher risk of losing their jobs and experiencing income drops. This practice increases the vulnerability of immigrants to poverty more significantly in Sweden, where the rule is institutionalized, than in Finland.

In addition, Sweden and Finland also differ in their employment protection legislation (EPL). A notable distinction lies in the so-called EPL gap, which reflects the differences in protection between regular and temporary workers. Sweden exhibits larger gaps, with temporary employment being significantly less protected than permanent employment. This creates a greater divide among workers in Sweden between, placing those with temporary contracts at a higher risk of being fired. Crucially, immigrants are more likely than the majority population to hold temporary contracts (Joona & Wadensjö, 2008). Temporary contracts not only entail greater job instability but also poorer working conditions, such as higher risks of overeducation and lower wages. As a result, immigrants in Sweden face greater exposure to job loss and lower incomes compared to the majority population.

A further dimension contributing to greater inequality in Sweden than Finland may be linked to differences in socio-demographic characteristics of the native-born and immigrant-born populations in the two countries. Additional analyses, presented in the Appendix (Table A1 and Table A2), show that socio-demographic characteristics which are associated with poverty risks are more prevalent among families with immigrant background in Sweden as compared to Finland. G2 children in Sweden are more likely to live in families where neither parent is employed and less likely to live in families where both parents are employed as compared to G2 children in Finland. At the same time, G2 children in Sweden are more likely to live with a single parent and to live in larger families than G2 children in Finland. Importantly, as said, these are well-established risk factors for poverty.

Our findings demonstrate very large inequalities with G2 children having longer exposure to poverty during early childhood. This, however, is not due to inequalities in poverty persistence, rather in the likelihood to fall into poverty. An important implication from this is that poverty entry rates likely explain much of the inequality we observe in other dimensions of poverty. In both countries, we find that G2 children are far more likely to fall into poverty than majority children. On the one hand, entry rates were observed to be higher in Finland as compared to Sweden while, on the other hand, persistence rates were the opposite. This might be related to differences in family policies where Sweden and Finland have been shown to differ in terms of mothers’ return to the labor market after parental leave, for example the Finnish home-care allowance system (Rønsen & Sundström, 2002). However, non-contributory child benefits are broadly similar in both countries and thus unlikely to explain these differences.

It is worth noting our study focuses on a specific phase in family life, the period just after the birth of a child, a life stage that is strongly influenced by the social policies targeted to parents and families with children. Childbirth strongly affects labor market attachment, earnings and reliance to social transfers among both immigrant and native parents. Accordingly, our own additional results (Table A3 in the Appendix) show that poverty rates are the highest when the child is below 1 year old, and decrease afterward. In other words, families are at a heightened risk of poverty when a child is born, and this is especially so among some immigrant groups. This could be explained by the level of benefits received: in the groups with the highest risk of poverty, mothers are likely to not be eligible for earnings-related parental leave benefits thereby reducing their income significantly after childbirth. The average background characteristics demonstrate that parents’ employment is significantly lower among G2 children. In Finland, majority mothers are twice as likely to be employed compared to immigrant mothers. This is common when women arrive to the host country through family reunification. As Vaalavuo and Rask (2024) mention, dividing immigrants to those actively seeking employment and those who are not (especially women) in the Finnish integration services might have long-lasting effects on families. Stronger support to access local labor market would be beneficial in increasing (future) mothers’ employment and could be the most effective way to reduce child poverty among G2. As employment history before childbirth affects the level of parental leave benefits and the chances of getting back to work, enhancing women’s labor market participation early on would be important.

This article provides a description of longitudinal poverty in two Nordic welfare states that are most highly ranked in terms of immigrant integration. This implies that inequalities registered in our study likely represent a lower bound estimate that can be used as a benchmark for future research focusing on countries characterized by less generous welfare states and less beneficial policy environment for immigrants.

Another venue for future research is investigating heterogeneity across mixed couples, in fact among some migrant groups intermarriage is a relatively common phenomenon which might modify the risk of childhood poverty. Additionally, future research should aim disentangle the causal mechanisms that put immigrant groups at a higher risk of experiencing multiple years of poverty as compared to the majority population. This means going beyond descriptively studying poverty and implies identifying how confounding and mediating factors explain the relationship between origin and poverty. Doing so will directly inform policy makers in how to design effective interventions to reduce inequality by breaking poverty cycles.

## Supplementary Information

Below is the link to the electronic supplementary material.

Supplementary file1 (DOCX 317 KB)

## Footnotes

1 Finnish data are maintained by Statistics Finland (permission number TK-53–1221-20) while Swedish data are made available by Statistics Sweden.

2 The lower childhood poverty rate in Finland than in Sweden is in line with existing recent research (Parolin and Filauro 2023; UNICEF Innocenti 2023).

3 The discrepancy between the snapshot poverty rates and poverty length of exposure is due to the fact that the former is calculated based on person-years while the latter refers to individuals. For example, in the case of the Somali population, 7.2 percent of individuals experience no poverty, whereas 79.2 percent of person-years are observed as poor.
