# Supplementary material for: Poverty Dynamics in Early Childhood among the Native-Born Children of Immigrants in Sweden and Finland
Source: Eur J Popul. 2026 Mar 25;42(1):13. doi: 10.1007/s10680-026-09772-y (PMC13048870; doi:10.1007/s10680-026-09772-y)
Supplement: Supplementary file 1 — Supplementary file1 (DOCX 317 KB) [file 10680_2026_9772_MOESM1_ESM.docx]

**Appendix/Supplementary information**

| **Figure A1.** Poverty gap: % distance from poverty cut-off for poor and non-poor children. |
| --- |
| 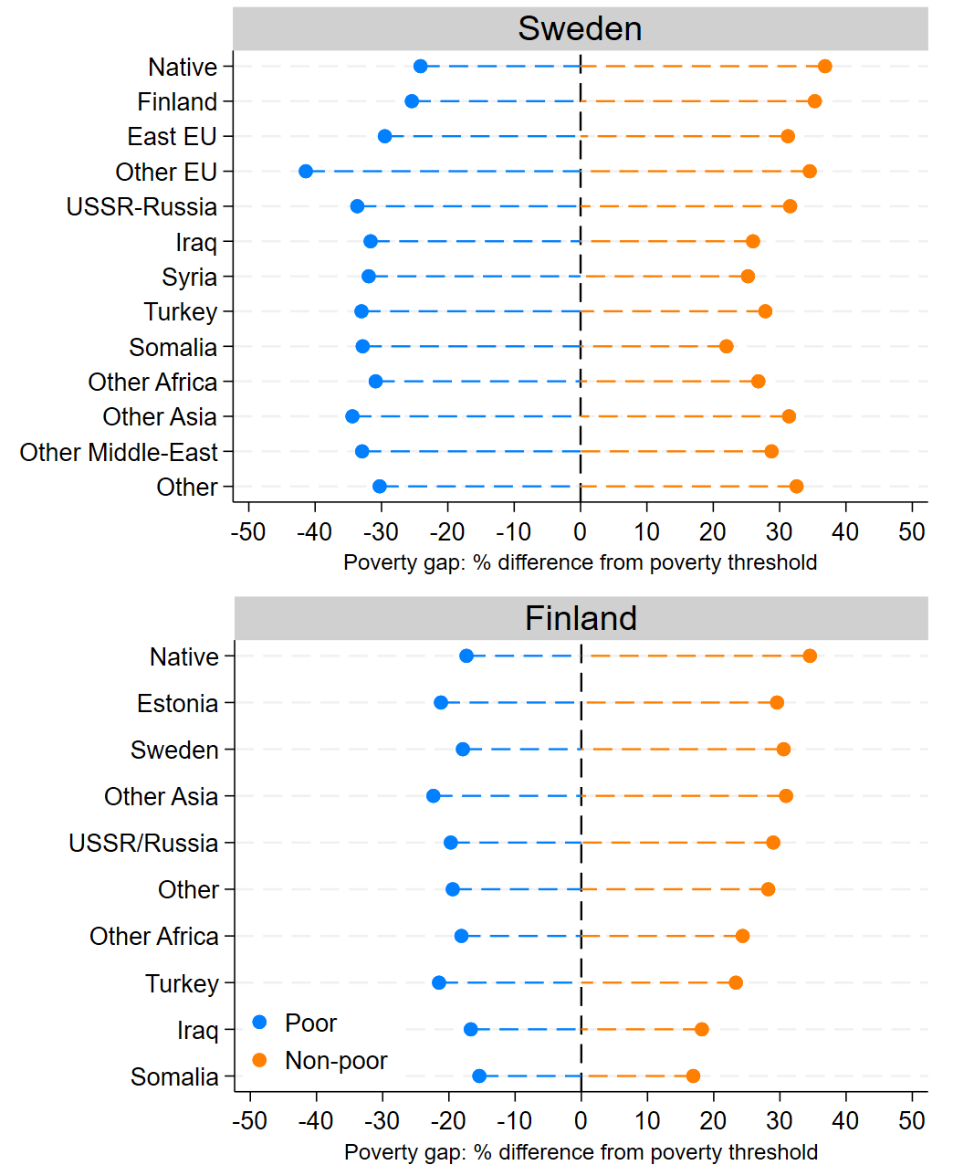 |

| **Table A1.** Socio-demographic composition of majority and G2 populations. Sweden. | | | | | | |
| --- | --- | --- | --- | --- | --- | --- |
|  | | | | | | |
| **N. of parents employed** | | | | |  |  |
|  | **None** | **One** | **Both** | **Total** |  |  |
| Native | 8.73 | 44.52 | 46.74 | 100 |  |  |
| G2 | 28.73 | 48.6 | 22.67 | 100 |  |  |
|  |  |  |  |  |  |  |
|  |  |  |  |  |  |  |
| **Parental partnership status** | | | |  |  |  |
|  | **Parents not together** | **Parents together** | **Total** |  |  |  |
| Native | 33.72 | 66.28 | 100 |  |  |  |
| G2 | 37.59 | 62.41 | 100 |  |  |  |
|  |  |  |  |  |  |  |
|  |  |  |  |  |  |  |
| **N. of other children in the household** | | | | | |  |
|  | **0** | **1** | **2** | **3+** | **Missing** | **Total** |
| Native | 30.44 | 49.4 | 15.72 | 4.43 | 0.01 | 100 |
| G2 | 28.72 | 40.6 | 18.67 | 11.82 | 0.2 | 100 |
|  |  |  |  |  |  |  |

| **Table A2.** Socio-demographic composition of majority and G2 populations. Finland. | | | | | |
| --- | --- | --- | --- | --- | --- |
|  | | | | | |
| **N. of parents employed** | | | | |  |
|  | **None** | **One** | **Both** | **Total** |  |
| Native | 6.18 | 28.83 | 64.99 | 100 |  |
| G2 | 24.06 | 45.94 | 30.00 | 100 |  |
|  |  |  |  |  |  |
|  |  |  |  |  |  |
| **Parental partnership status** | | | |  |  |
|  | **Parents not together** | **Parents together** | **Total** |  |  |
| Native | 11.38 | 88.62 | 100 |  |  |
| G2 | 18.76 | 81.24 | 100 |  |  |
|  |  |  |  |  |  |
|  |  |  |  |  |  |
| **N. of other children in the household** | | | | | |
|  | **0** | **1** | **2** | **3+** | **Total** |
| Native | 28.14 | 42.60 | 18.10 | 11.17 | 100 |
| G2 | 32.18 | 40.25 | 16.97 | 10.60 | 100 |

| **Table A3.** Poverty rate by age, Sweden and Finland | | | | | |
| --- | --- | --- | --- | --- | --- |
| **Sweden** | **Age** | | | | |
|  | **0** | **1** | **2** | **3** | **4** |
| Native | 12.75 | 11.7 | 9.68 | 9.06 | 9.34 |
| Finland | 15.48 | 13.92 | 12.51 | 11.51 | 11.16 |
| Other EU | 25.99 | 24.24 | 22.15 | 19.87 | 19.51 |
| East EU | 37.62 | 33.63 | 29.3 | 26.15 | 25.08 |
| USSR-Russia | 38.89 | 35.29 | 32.56 | 28.37 | 27.53 |
| Syria | 72.72 | 68.09 | 63.08 | 58.65 | 58.26 |
| Iraq | 69.42 | 65.71 | 61.21 | 55.73 | 54.03 |
| Turkey | 54.92 | 53.32 | 47.54 | 42.69 | 41.79 |
| Other Middle-East | 54.76 | 51.33 | 46.83 | 42.98 | 42.11 |
| Other Asia | 35.43 | 32.1 | 30.28 | 27.1 | 26.7 |
| Somalia | 83.45 | 81.02 | 78.23 | 76.28 | 76.81 |
| Other Africa | 59.81 | 55.34 | 50.56 | 46.51 | 45.35 |
| Other | 28.52 | 25.32 | 22.87 | 19.71 | 18.9 |
| **Total** | **22.23** | **20.57** | **18.15** | **16.72** | **16.69** |
|  |  |  |  |  |  |
| **Finland** | **Age** | | | | |
|  | **0** | **1** | **2** | **3** | **4** |
| Finland | 13.27 | 12.48 | 11.89 | 10.47 | 9.69 |
| Sweden | 24.35 | 22.86 | 21.04 | 20.00 | 19.32 |
| Estonia | 28.23 | 25.73 | 25.39 | 20.87 | 18.16 |
| Other Asia | 25.63 | 23.26 | 23.09 | 21.58 | 19.23 |
| Other Africa | 43.24 | 35.02 | 33.24 | 32.13 | 30.95 |
| USSR/Russia | 31.46 | 26.37 | 25.37 | 22.61 | 20.49 |
| Somalia | 65.00 | 51.51 | 49.64 | 46.53 | 44.93 |
| Turkey | 58.61 | 51.37 | 50.64 | 46.12 | 40.03 |
| Iraq | 67.83 | 55.12 | 49.08 | 44.45 | 39.69 |
| Other | 33.11 | 28.15 | 25.81 | 24.00 | 21.77 |
| **Total** | **15.50** | **14.27** | **13.60** | **12.07** | **11.13** |
